# Outcomes after ^90^Yttrium-ibritumomab tiuxetan-BEAM in diffuse large B-cell lymphoma: a meta-analysis

**DOI:** 10.1002/cam4.247

**Published:** 2014-04-16

**Authors:** Sophie Auger-Quittet, Yohan Duny, Jean-Pierre Daures, Philipe Quittet

**Affiliations:** 1 Department of Internal Medicine, Mutualist Clinic BeausoleilMontpellier, France; 2 Department of Biostatistical and Epidemiology, INSERM Unit EAMontpellier, France; 3 Department of Hematology, University Hospital Saint-EloiMontpellier, France

**Keywords:** ^90^Y-Ibritumomab tiuxetan, autologous transplantation, DLBCL, meta-analysis, radioimmunotherapy, Z-BEAM

## Abstract

High-dose chemotherapy followed by autologous stem cell transplantation (ASCT) is a standard therapy in patients with relapsed/refractory diffuse large B-cell lymphoma (DLBCL) who are chemosensitive. The combination of carmustine, etoposide, cytarabine, and melphalan (BEAM) is commonly used as a conditioning regimen. The addition of yttrium-90 (^90^Y)-ibritumomab tiuxetan (Zevalin®) to BEAM (Z-BEAM) is increasingly being used to improve outcomes and overcome refractory disease. We conducted a literature review and meta-analysis in order to evaluate the clinical effects of Z-BEAM followed by ASCT in patients with DLBCL. A literature search was conducted for randomized controlled trials and observational studies of Z-BEAM as a conditioning regimen for ASCT in adult patients with DLBCL. Extracted data included baseline patient demographics, overall response (ORR), complete response (CR), overall survival (OS), progression-free survival (PFS), nonrelapse mortality (NRM), median time to ANC and platelet engraftment, and rate of myelodysplastic syndrome. Mixed-effects models were used to determine estimates. Ten studies (*N* = 328) were included in the meta-analysis. The 2-year OS and PFS were 84.5% (*n* = 328) and 67.2% (*n* = 285), respectively. Outcomes were superior in patients with nontransformed lymphoma. Posttransplant, ORR and CR rates were 72.6% and 68.5%, respectively. The NRM rate was 6.3% and the incidence rate of myelodysplastic syndrome was 2.5%. Two-year OS was significantly associated with pretransplant ORR (*P* = 0.008, *τ*^2^ = 0). There was no significant association between PFS and pretransplant response. Z-BEAM is safe and effective as a conditioning regimen in relapsed/refractory DLBCL.

## Introduction

High-dose chemotherapy followed by autologous transplantation (ASCT) is a standard treatment option for patients with chemotherapy-sensitive relapsed/refractory diffuse large B-cell lymphoma (DLBCL). In the PARMA study, Philip et al. found a 5-year event-free survival (EFS) rate of 46% in patients with chemotherapy-sensitive non-Hodgkin's lymphoma (NHL) who received high-dose chemotherapy and ASCT compared with 12% who received conventional chemotherapy 1.

Subsequent to publication of the PARMA study, the use of rituximab, an anti-CD20 monoclonal antibody, has improved response and survival in patients with DLBCL 2,3. Rituximab in combination with chemotherapy as salvage therapy remains effective for patients with relapsed and refractory DLBCL 4,5. A high-dose chemotherapy regimen combining carmustine, etoposide, cytarabine, and melphalan (BEAM), showed a 3-year progression-free survival (PFS) rate of 53% 5. However, relapse continues to be the primary cause of treatment failure in patients with NHL undergoing high-dose therapy and ASCT. DLBCLs are known to be radiosensitive, however, total body irradiation (TBI) is associated with substantial morbidity 6. The way to optimize conditioning regimens with radiolabeled antibody delivering radiation of total body with less toxicity could be radioimmunotherapy.

Radioimmunotherapy with ^90^Y-ibritumomab tiuxetan (Zevalin®) is effective in indolent lymphomas but is also being studied in DLBCL 7,8. In 2007, the first report of ^90^Y-ibritumomab tiuxetan combined with BEAM followed by ASCT (Z-BEAM) in 23 patients with chemotherapy refractory aggressive lymphoma showed that the regimen improved outcome and overcame chemotherapy refractory status 9. Despite these encouraging results, the 2-year cumulative incidence of relapse was 31% and the estimated 2-year OS was 67%. In a randomized study comparing Z-BEAM to BEAM prior to ASCT in patients with relapsed aggressive lymphoma, a significant 2-year OS survival benefit was observed with Z-BEAM, but there was no difference in 2-year PFS between the two regimens 10. Additional studies have been published reporting the safety and efficacy of Z-BEAM in B-cell lymphomas. However, the majority of reports had heterogeneous B-cell lymphoma types including DLBCL, transformed follicular lymphoma, mantle cell lymphoma, or Richter's syndrome. In addition to, not all patients were chemosensitive prior to ASCT.

Randomized trials are ongoing of Z-BEAM followed by ASCT in DLBCL. Although preliminary results are promising, it is important to further elucidate the regimen's safety and efficacy. Therefore, we performed the first noncomparative meta-analysis of randomized trials, prospective, and observational studies on the effect of Z-BEAM followed by ASCT in patients with DLBCL.

## Methods

### Data sources and searches

Overall methods were adapted from MOOS and PRISMA guidelines for meta-analyses 11,12. A literature search was conducted to identify randomized controlled trials (RCTs) and observational studies of ^90^Y-ibritumomab tiuxetan + BEAM (Z-BEAM) and autologous stem cell transplantation in adult DLBCL. On 30 June 2013, a systematic literature search was performed on the following databases: the National Library of Medicine PubMed (http://www.ncbi.nlm.nih.gov/pubmed), the American Society of Hematology (http://www.hematologylibrary.org), the European Haematology Association (http://www.haematologica.org), and the American Society of Clinical Oncology (http://meetinglibrary.asco.org/abstracts). The search terms were B-cell lymphoma [MeSH] and Zevalin® (Spectrum Pharmaceuticals Inc., Henderson, NV) or yttrium-90 (^90^Y)-ibritumomab tiuxetan and Z-BEAM. The results were limited to adult human studies reported in the English or French language.

### Study selection

The literature searches, study selection, and data extraction were carried out independently by two investigators (S. A., P. Q.), discrepancies were solved by discussion until consensus was reached. Studies were included in the meta-analysis if they matched all prespecified eligibility criteria. Studies were excluded for the following reasons: (1) lymphoma other than DLBCL (e.g., mantle cell lymphoma, Richter syndrome, or indolent lymphoma [except for transformed follicular lymphoma]), (2) age <18 years, (3) consolidation without ASCT, and (4) allogeneic stem cell transplantation. Studies with patient populations of mixed lymphomas were included if the required data for patients with DLBCL were extractable from the reports. Finally, studies enrolling fewer than five patients were not included due to risk of extreme bias. Reviews and registers were not included. There was no restriction in study design and reports in abstract form were included. When updated or duplicated patient population analyses were found, only the most recent publications were retained. For the initial screening, titles and abstracts of all articles were scrutinized for relevancy. Then, the full text of each retained article was analyzed for whether it met the prespecified eligibility criteria.

### Data extraction and quality assessment

From eligible studies, two investigators independently extracted data using designated forms. Between-study variables included information on study characteristics (author(s), publication year, journal, study design, follow-up duration) and sample characteristics (country, stage of lymphoma, International Prognostic Index [IPI], incidence of transformed lymphoma, number of prior treatments, use of rituximab in prior therapy, sample size). For each study, baseline demographics (mean age, gender), response data (ORR, CR, OS, and PFS), and toxicity data (NRM, median time to ANC engraftment, median time to platelet engraftment, rate of myelodysplastic syndrome) after RIT ACST were extracted if available. Individual data were used where possible. Classical tools (Newcastle-Ottawa Scale [NOS] or Jadad scale) to assess the quality of studies were not utilized in all included studies; therefore, the investigators chose a quality assessment score on compounded items extracted from classical quality assessment scales (type of publication, years, study design, randomization). This quality assessment is detailed in Table4. Study quality was reported, independently in a blind study by, author, journal, year of publication, and results by both reviewers.

### Data synthesis and analysis

The primary assessment for this study was OS and PFS at 2 years. Secondary assessments were 3-year OS, 3-year PFS, ORR, CR rate, incidence of myelodysplastic syndrome, and NRM. For OS and PFS, mean values were estimated by taking into account censored data. Secondary analyses included estimation of OS and PFS at 2 years in prospective studies according to DLBCL and transformed lymphoma.

We determined estimates of the effect of Z-BEAM followed by ASCT by calculating the weighted median proportion (event rate). To account for expected heterogeneity, the mixed-effects model was used. Summary estimates of event rates were obtained using the fixed-effects model method when heterogeneity was not significant. The percentage of variability beyond chance was estimated using the *I*² statistic 13,14. An *I*² statistic for which values are over 50% (*P* < 0.1) may indicate substantial heterogeneity. To explain heterogeneity, the effects of quantitative covariates on study event rates were investigated using mixed-effects metaregressions (method of moments). Qualitative covariates were investigated using mixed-effects analysis.

The risk of publication bias was assessed using the one-tailed Egger's test and graphically by the funnel plot, which plots the natural logit of study risk ratios versus their standard error 15. Additionally, Duval and Tweedie's trim-and-fill method was used 16. A *P*-value below 0.05 was considered statistically significant in all analysis. In sensitivity analyses, the impact of study selection was addressed by a “one-study removed” meta-analysis approach.

Finally, we performed a sub meta-analysis to compare OS at 2 years between BEAM and Z-BEAM. We used studies comparing Z-BEAM and BEAM.

All analyses were performed using comprehensive meta-analysis (Comprehensive Meta-Analysis, Version 2.2.048, 7 November 2008; Biostat, 14 North Dean Street, Englewood, NJ) and STATA (Stata/SE 12.1 for Windows Revision, 9 July 2013, Copyright 1985–2011, StataCorp LP 4905, Lakeway Drive, College Station, TX).

## Results

### Study selection and description

**Figure 1:**
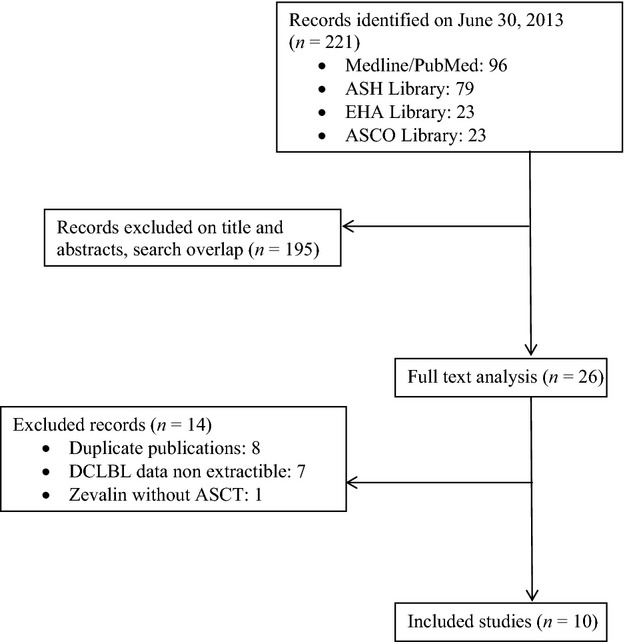
Flow diagram of the trial selection process.

A flow diagram of the search results is presented in Figure1. A total of 221 records were identified through electronic databases. Removing search overlap and irrelevant studies based on title and abstract review reduced the results to 26 records. After full-text analysis, 10 studies were chosen for inclusion in the meta-analysis (five abstracts, four articles, one letter). Four of the studies were prospective, multicenter studies 9,10,17,18 and six studies were retrospective 6,19–23. None of the studies contained individual data. The studies were classified according to their design and year of publication.

Study characteristics, including design, populations, and outcomes are presented in Table1. Across the 10 studies, 328 patients with DLBCL received ^90^Y-ibritumomab tiuxetan followed by ASCT. ^90^Y-ibritumomab tiuxetan was generally administered by intravenous injection at 0.3 or 0.4 mCi/kg according to platelet count and followed by BEAM. In one study, patients received etoposide and cyclophosphamide 18.

**Table 1:** Study characteristics.

Reference	Article type	Prospective design	N	Median age (years)	Female (%)	Transformed lymphoma (%)	2-year OS (%)	2-year PFS (%)	ORR (%)	CR rate (%)	Median day of ANC >0.5 × 10^9^/L	Median day of platelet > 20 × 10^9^/L	Median day of platelet > 50 × 10^9^/L	NRM rate (%)	MDS rate (%)
Siddiqi et al. 19	Abstract	N	36	54.5	33	0	79	64	–	–	–	–	–	–	–
Wondergem et al. 20	Abstract	N	43	56	–	0	90	–	–	–	–	–	–	0	–
Shimoni et al. 10	Article	Y	22	58	27.2	27.2	91	59	–	95	10	13	–	4.5	0
Alousi et al. 21	Abstract	N	25	–	–	0	92	84	–	–	9	–	–	4	–
Briones et al. 17	Abstract	Y	30	53	–	26.6	65	63	70	60	11	13	–	13.3	3.3
Nademanee et al. 22	Abstract	N	62	53	–	–	91	74	–	–	–	–	–	–	–
Nademanee 18	Article	Y	18	51	45	0	93	74	–	–	10	12	19	3	–
Shimoni et al. 9	Article	Y	22	55	40.9	27.2	67	52	90	50	10	12	–	9	–
Krishnan et al. 6	Article	N	46	56.5	37	0	81	61	63	–	–	–	–	0	2.1
Wondergem et al. 23	Letter	N	24	–	–	100	100	80	–	–	15	15	24	0	–

ANC, absolute neutrophil count; CR, complete response; MDS, myelodysplastic syndrome; NRM, nonrelapse mortality; ORR, objective response rate; OS, overall survival, PFS, progression-free survival.

### Overall survival and progression-free survival

**Figure 2:**
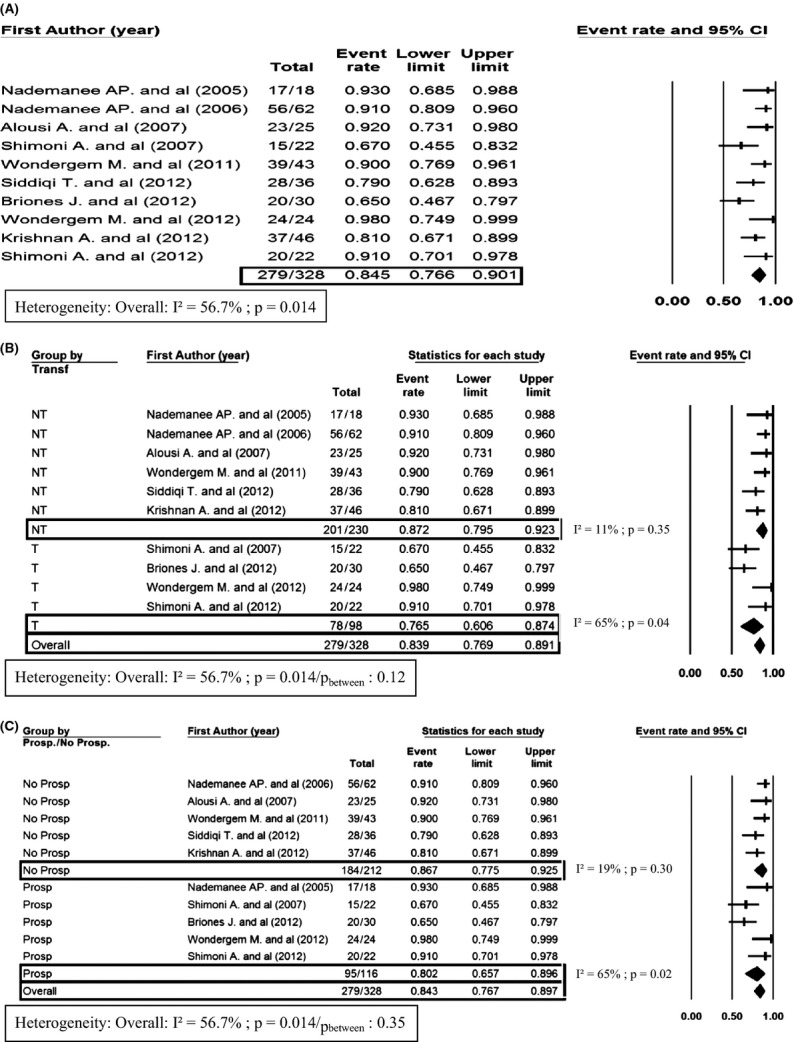
Forest plot of OS at 2 years (A), OS at 2 years defined by transformed (T) or not transformed (NT) (B), OS at 2 years defined by prospective (Prosp) or not prospective (No Prosp) studies (C). Heterogeneity is estimated with *I*² statistic according to Higgins et al. 13 and Higgins and Thompson 14. By group analysis for both NT/T and Prosp/No prosp is made by mixed-effects analysis. OS, overall survival.

In 328 patients who were available for OS analysis, the 2-year OS was 84.5% (95% CI, 76.6–90.1) (Fig.2A). Heterogeneity was high (*I*² > 50%, *P* = 0.014). In patients without transformed lymphoma, 2-year OS was 87.2% (95% CI, 79.5–92.3) versus 76.5% (95% CI, 60.6–87.4) in the transformed lymphoma group (Fig.2B). There was no significant heterogeneity between transformed and nontransformed groups (*P* = 0.12). In prospective studies, 2-year OS was 80.2% (95% CI, 65.7–89.6) versus 86.7% (95% CI, 77.5–92.5) in retrospective studies (Fig.2C). There was no significant heterogeneity between prospective and retrospective groups (*P* = 0.35).

**Figure 3:**
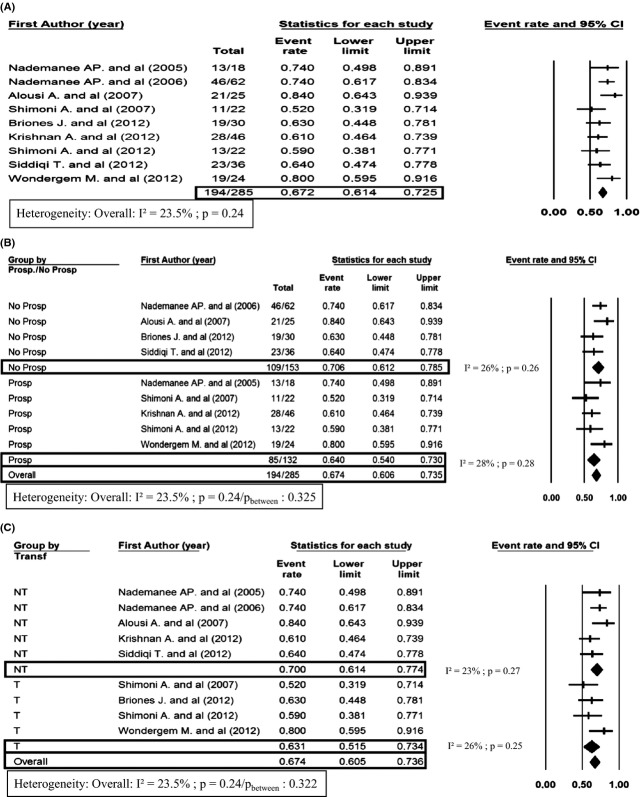
Forest plot of PFS at 2 years (A), PFS at 2 years defined by transformed (T) or not transformed (NT) (B), PFS at 2 years defined by prospective (Prosp) or not prospective (No Prosp) studies (C). Heterogeneity is estimated with I² statistic according to Higgins et al. 13 and Higgins and Thompson 14. By group analysis for both NT/T and Prosp/No prosp is made by fixed-effects analysis. OS, overall survival; PFS, progression-free survival.

In 285 patients who were available for PFS analysis, the 2-year PFS was 67.2% (95% CI, 61.4–72.5) with nonsignificant heterogeneity among studies (*I*² < 10%, *P* = 0.24) (Fig.3A). In patients without transformed lymphoma, 2-year PFS was 70% (95% CI, 61.4–77.4) versus 63.1% (95% CI, 51.5–73.4) in the transformed lymphoma group (Fig.3C). There was no heterogeneity between the groups (*P* = 0.32). In prospective studies, 2-year PFS was 64% (95% CI, 54.0–73.0) versus 70.6% (95% CI, 61.2–78.5) in retrospective studies (Fig.3B) with no significant heterogeneity between the two groups (*P* = 0.325).

At 3 years, OS and PFS were 85.8% (95% CI, 78.4–91) and 61.6% (95% CI, 49.2–72.6) for 129 and 64 patients, respectively. Heterogeneity was null for both (*I*^2^ = 0, *P* = 0.53 and 0.88, respectively).

### Response and safety

The posttransplant ORR was 72.6% (95% CI, 55.5–85) in 98 patients for whom data were available. Heterogeneity was high (*I*² < 50%, *P* = 0.098). The CR rate was 68.5% (95% CI, 40.5–87.5) in 74 patients for whom data were available. Heterogeneity was high (*I*² > 50%, *P* = 0.022). The rate of relapse was 31.8% (95% CI, 24.2–40.5) for 128 patients with no significant heterogeneity.

The NRM rate was 6.3% (95% CI, 3.4–11.4) for 230 patients, with no significant heterogeneity (*I*² = 2.5%, *P* = 0.41). Death were related to sepsis (*n* = 3), cerebral hemorrhage (*n* = 1), second malignancies (*n* = 1) (LAM), multiorgan toxicities (*n* = 2), and in two studies causes of death are not given.

The incidence rate of myelodysplastic syndrome was 2.5% (95% CI, 0.7–8.5) for 98 patients in three studies.

The median day of ANC engraftment was between D9 and D15, the median day for platelet count higher than 20 × 10^9^/L was between D12 and D15, and over 50 × 10^9^/L was between D19 and D24 (data were only available from two publications).

Comparison between Z-BEAM and BEAM was performed with three studies 10,20,21. Outcomes with Z-BEAM were better than BEAM for OS at 2 years (HR = 4.09; 95% CI, 2.01–8.3; *P* < 0.001) and for PFS at 2 years (HR = 1.97; 95% CI, 1.04–3.72; *P* = 0.036). There was no heterogeneity (*I*^2^ = 0, *P* = 0.52 and 0.96, respectively) (Fig.7A and B). Egger's test showed no evidence of publication bias of studies on OS and PFS at 2 years. This is consistent with the shape of the funnel plot showing good symmetry.

### Investigating heterogeneity

**Figure 4:**
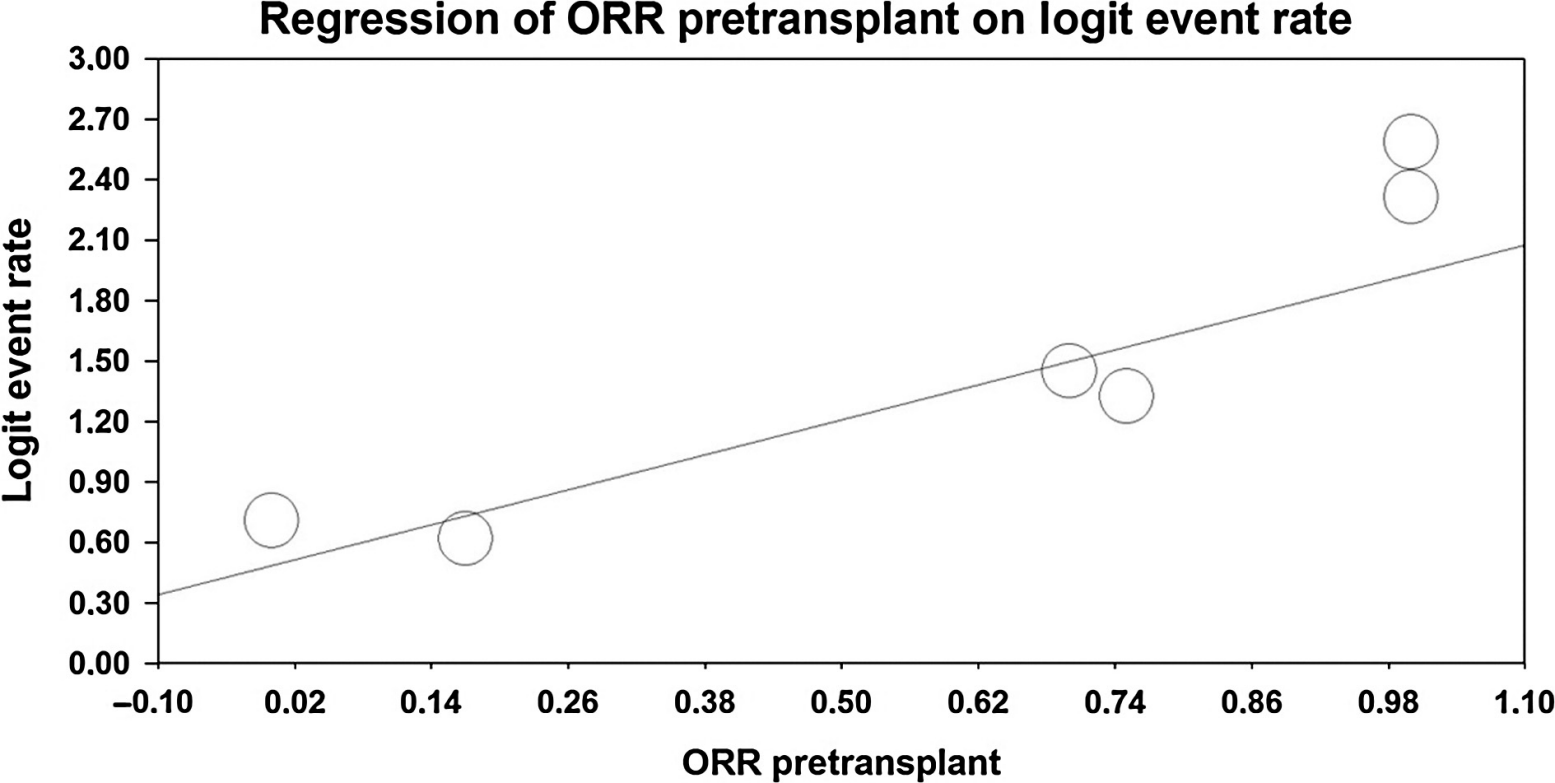
Measured (circle) and predicted (line) 2-year OS depending on the difference in pretransplant ORR in mixed-effects metaregression analysis. ORR, overall response; OS, overall survival.

In an univariate mixed-effects metaregression analysis (including year of publication, age, sex, percentage of transformed lymphoma in sample size, pretransplant ORR), 2-year OS was significantly associated with differences in pretransplant ORR (*P* = 0.008, *τ*^2^ = 0) (Fig.4). In other words, patients with superior pretransplant response were more likely to be alive at 2 years. None of the other variables were significantly correlated with 2-year OS (Table2). None of the variables analyzed were significantly correlated with 2-year PFS (Table3). Number of data for CR were too small to include this feature in metaregression.

**Table 2:** Results of mixed-effects metaregression on 2-year OS.

Moderator	Study *N*	Slope	Slope 95% CI	*P*-value	*τ*^2^
Publication year	10	−0.07	−0.26, 0.12	0.44	0.37
Age	8	0.02	−0.28, 0.32	0.88	0.38
Female%	5	−0.04	−0.14, 0.06	0.41	0.15
Transformed%	9	−0.007	−0.02, 0.01	0.46	0.38
Pretransplant. ORR	6	1.45	0.38, 2.52	0.008	0

ORR, overall response rate; OS, overall survival.

**Table 3:** Results of mixed-effects metaregression on 2-year PFS.

Moderator	Study *N*	Slope	Slope 95% CI	*P*-value	*τ*^2^
Publication year	9	−0.06	−0.15, 0.04	0.25	0.04
Age	7	−0.11	−0.25, 0.02	0.12	0
Female (%)	5	0.01	−0.05, 0.07	0.75	0
Transformed (%)	8	0.004	−0.006, 0.01	0.43	0.06
Pretransplant. ORR	6	0.36	−0.52, 1.24	0.42	0

ORR, overall response rate; PFS, progression-free survival.

We could not identify any associations between trial quality and ORR or CR using univariate mixed-effects metaregression analysis. Using a prospective or retrospective design and abstract or article was not significantly associated with response rate.

With a “one-study removed” approach, 2-year OS ranged from 83.2% to 86.1% around 84.5% and 2-year PFS ranged from 65.4% to 68.6% around 67.2%. The impact of each study was equivalent on these parameters.

### Publication bias

**Figure 5:**
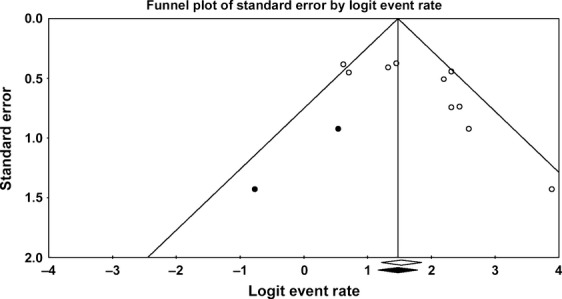
Funnel plot of natural logarithm of 2-year OS. Results are based on the 10 included studies for 2-year OS (open circles) and with the addition of three virtual studies identified by “trim-and-fill” analysis (black circles) (Egger et al. 15, Duval and Tweedie, 16). OS, overall survival.

Results of an Egger's test indicated a publication bias of studies on 2-year OS (*P* = 0.01), 3-year OS (*P* = 0.09), posttransplant ORR (*P* = 0.02), NRM (*P* < 0.001), and relapse rate (*P* = 0.014). The publication bias found on 2-year OS is consistent with the asymmetrical shape of the funnel plot observed in Figure5. There were three missing studies for 2-year OS. After adding the three missing studies by utilizing the “trim-and-fill” method, a trend of reduced 2-year OS was observed (83%; 95% CI, 77–89). There were two missing studies for 3-year OS and two for posttransplant ORR showing a lower event rate. Adding the missing studies via the trim-and-fill method reduced 3-year OS (82.4%; 95% CI, 75.1–87.9) and posttransplant ORR (63%; 95% CI, 54–71). There were four missing studies for NRM and one missing study for relapse rate showing a higher event rate. Adding the missing studies via the trim-and-fill method increased NRM (8.9%; 95% CI, 5.2–15.0) and relapse rate (34%; 95% CI, 26–43). Concerning 3-year PFS and CR, the funnel plot shape showed symmetry.

**Figure 6:**
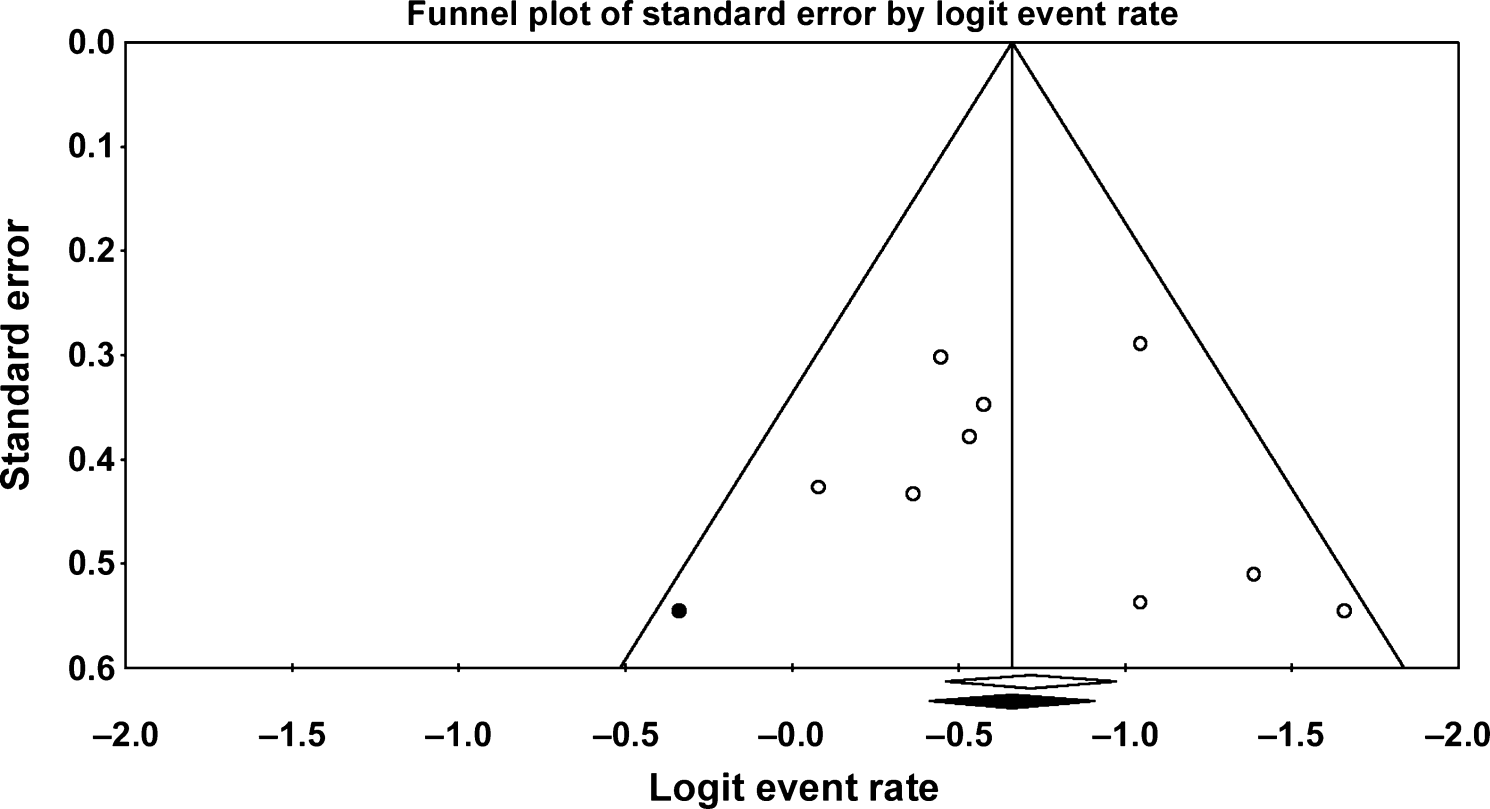
Funnel plot of natural logarithm of 2-year PFS. Results are based on the nine included studies for 2-year PFS (open circles) and with the addition of one virtual study identified by “trim-and-fill” analysis (black circles) (Egger et al. 15, Duval and Tweedie 16). PFS, progression-free survival.

**Figure 7:**
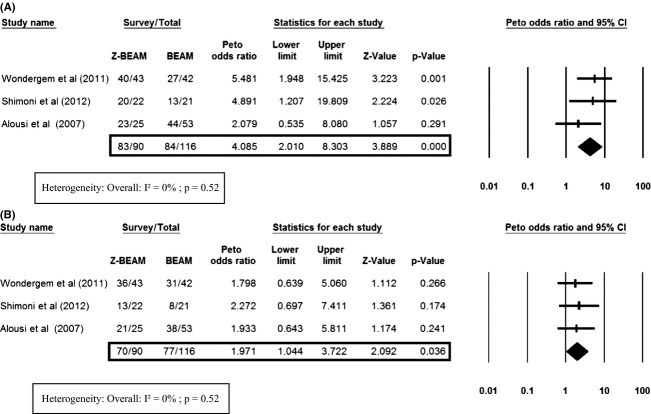
(A) Forest plot of OS at 2 years. Meta-analysis for trials comparing BEAM with Z-BEAM under fixed method. (B) Forest plot of PFS at 2 years. Meta-analysis for trials comparing BEAM with Z-BEAM under fixed method. Heterogeneity is estimated with *I*² statistic according to Higgins et al. 13 and Higgins and Thompson 14. OS, overall survival; PFS, progression-free survival.

For 2-year PFS, the Egger's test showed no evidence of publication bias (*P* = 0.20); this is consistent with the symmetrical shape of the funnel plot. There was one missing study for 2-year PFS. Adding the study by using the trim-and-fill method reduced the 2-year PFS (65.9%; 95% CI, 60.2–71.2) (Fig.6).

## Discussion

This report is, to our knowledge, the first to show in a large sample of patients, the clinical outcomes of ^90^Y-ibritumomab tiuxetan combined with BEAM (Z-BEAM) followed by ASCT in patients with DLBCL. Our goal is to highlight ^90^Y-ibritumomab tiuxetan's place in ASCT regimen. However, the results of this meta-analysis are to be interpreted as descriptive and not comparative. Relatively few trials of Z-BEAM prior to ASCT in DLBCL have been described in the literature to date. Many of the studies are retrospective in design or utilize a historical matched control with only one randomized study designed to directly compare the Z-BEAM regimen with BEAM 10. Moreover, some reports have only been published in abstract form and did not provide sufficient details to be used in this meta-analysis or the data for DLBCL patients was often not extractable.

In our report, Z-BEAM in patients with DLBCL resulted in a 2-year OS of 84.5% and a 2-year PFS of 67.2% with a NRM of 6.3%. In the prerituximab era, the PARMA study showed a 5-year OS rate of 53% using BEAC (carmustine, etoposide, cytarabine, cyclophosphamide) as conditioning regimen with or without radiotherapy 1. Currently, with the standard use of rituximab in B-cell malignancies, BEAM is considered the standard regimen for patients with relapsed NHL. Gisselbrecht showed a 3-year PFS of 39% and a 3-year OS of 53% in DLBCL 5. In a registry study of the European Society for Blood and Marrow Transplantation (EBMT), 5-year OS was 60% and 5-year disease-free survival (DFS) was 44% for patients with DLBCL who received the BEAM regimen and ASCT 24. Results of these studies show that outcomes need to be improved. In this way, in 2012, Shimoni et al. reported a 2-year OS of 91% versus 62% (*P* = 0.05) for Z-BEAM and BEAM, respectively, in a randomized trial comparing the two regimens as conditioning prior to ASCT in chemosensitive patients with relapsed aggressive lymphoma 10. However, no significant difference was observed for 2-year PFS between the regimens.

It has been previously demonstrated that the best outcomes were achieved in patients who were chemosensitive prior to ASCT 1,25. However, encouraging results were observed with the Z-BEAM regimen in chemorefractory patients with a 2-year OS of 67% 9. While chemosensitivity is an important advantage for patients who receive BEAM, the Z-BEAM regimen may convert a patient's refractory status to sensitive 9.

Chemorefractory patients were present in our selected studies which further emphasize the importance of our 2-year OS and PFS findings. In our analysis, 2-year OS was significantly associated with pretransplant ORR (*P* = 0.002, *τ*^2^ = 0). None of our measured parameters were significantly correlated with 2-year PFS. This is potentially due to a lack of heterogeneity or because Z-BEAM therapy overcame a poor pretransplant response rate and improved PFS. Shimoni showed similar results in univariate analyses according to prognostic factors: no significant difference in response status at ASCT, but a significant difference according to the conditioning regimen of BEAM or Z-BEAM. The results further emphasize the increase efficacy observed with the Z-BEAM regimen 10. Our results were comparable with other literature concluding that adding radioimmunotherapy to the BEAM conditioning regimen could improve OS.

Knowing the responsiveness of NHL to radiation in the ASCT regimen is essential. Before BEAM, total body irradiation (TBI) or radiotherapy in the involved area was used. Limitations of TBI included the toxicity resulting from irradiating normal tissues as well as the inability to deliver higher doses to areas of tumor involvement beyond the background uniform TBI dose (typically in the range 12–15.75 Gy). The dose of radiation delivered by radioimmunotherapy to the tumor is 10-fold higher than that possible with TBI leading to better outcomes as shown by a comparative study in which 4-year OS with Z-BEAM was 81% versus 52.7% for TBI combined with cyclophosphamide and etoposide 6. Furthermore, toxicity with Z-BEAM was similar to that associated with BEAM alone and lower than TBI with a NRM of 0% in the Z-BEAM group compared to 15.8% for the TBI group at 4 years 6,26. In our study, the NRM was 6.3%.

Utilizing data from four prospective studies (including one phase III trial) among the 10 analyzed studies gives strength to this meta-analysis. Formal meta-analytic methods can only use RCTs. Currently, there is a lack of RCTs regarding the use of ^90^Y-ibritumomab tiuxetan and ASCT in patients with DLBCL. We used rigorous methods to analyze and synthesize the evidence for ^90^Y-ibritumomab tiuxetan efficacy in DLBCL. This meta-analysis used a large population that was not limited to RCTs. However, none of the studies had a particular impact, as shown by the “one-study removed” approach. Analysis of bias of publication revealed no evidence of lack of study for 2-year PFS but a lack of three studies for 2-year OS. Therefore, utilizing the trim-and-fill method resulted in a 2-year OS of 83%.

The heterogeneity in the design of the studies and the type of lymphoma may be a limitation in interpreting the results of this study. While, in all subanalyses, the 2-year OS and 2-year PFS were stable, they were found to be higher in the subanalysis of retrospective studies (86.7% and 70.6%, respectively) than prospective studies. As expected, better outcomes were observed in DLBCL (OS 87.2% and PFS 70%) in comparison to transformed lymphoma (76.5% and 63.1%). There was no heterogeneity between both groups. The heterogeneity (*I*^2^) of the various analyses was taken into account by the use of a random model. Patients were heterogeneous in regard to age, sex, previous treatment, and year of publication. We could not explore previous treatment because data were not reported separately. Unfortunately, dominant poor prognosis factors as secondary high IPI, early relapse, and positive PET-CT at ASCT were available (given or extractible) only in three studies and have not been studied 10. Yet, they could influence heterogeneity. Almost all patients (not in only one study, unknown in two studies) received rituximab and this factor has not been studied too. However, we can assume that patients having not received rituximab in front line, would probably have still anti-CD20 sensibility. Hence they could be more sensitive to ^90^Y-ibritumomab tiuxetan. In the same way, pretransplant CR may be over estimated by the lack of PET-CT.

Surprisingly, there is no effect on transformed rate in metaregression. Our 2-year OS findings could be up-estimated due to bias of publication.

Interpretations for 3-year OS and PFS (85.8% and 61.6%, respectively) should be cautious due to a smaller sample size; cautious interpretation is also recommended for myelodysplastic syndrome rate. However, our relative sample size comparable to other literature is quite high and is higher than those results published by BEAM alone 5. In this study, the rate of myelodysplastic syndrome was around 2.5% and comparable to what is shown in the literature 27.

As shown in submeta-analysis comparing BEAM and Z-BEAM, patients with Z-BEAM have better OS and PFS at 2 years (*P* < 0.01 and *P* < 0.05) than those receive BEAM. However, interpretation of submeta-analysis on three studies comparing BEAM and Z-BEAM should be cautious. There were only three studies and only a phase III trial, two retrospective studies with one comparison between BEAM and R-BEAM. However, results were and remained significant when this last study was removed (data not shown).

To conclude, ^90^Y-ibritumomab tiuxetan combined with BEAM as a conditioning regimen followed by ASCT produces a high rate of 2-year OS and 2-year PFS. NRM is moderate and similar to what is observed with BEAM alone. If 2-year OS remains significantly associated with pretransplant response, then ^90^Y-ibritumomab tiuxetan could overcome a poor pretransplant response rate, but randomized multicenter trial is needed to assess this observation before Z-BEAM regimen can be accepted as a standard.

## Conflict of Interest

None declared.

## Appendix

**A1:** Quality assessment score.

Reference	Country	Year of publication	Type of publication	Support of publication	Design	Randomized
Siddiqi et al. 19	USA	2012	Abstract	ASCO	Retrospective	N
Wondergem et al. 20	Netherlands	2011	Abstract	ASH	Retrospective, historical comparison	N
Shimoni et al. 10	Israel	2012	Article	Cancer	Prospective, multicentric	Y
Alousi et al. 21	USA	2007	Abstract	ASH	Retrospective, historical comparison	N
Briones et al. 17	Spain	2012	Abstract	ASH	Phase II, multicentric, prospective	N
Nademanee et al. 22	USA	2006	Abstract	ASH	Retrospective, historical comparison	N
Nademanee et al. 18	USA	2005	Article	Blood	Phase I/II, prospective, multicentric	N
Shimoni et al. 9	Israel	2007	Article	Exp Hematol	Phase II, prospective	N
Krishnan et al. 6	USA	2012	Article	Biol BMT	Retrospective, matched historical comparison	N
Wondergem et al. 23	Netherlands	2012	Letter	BJH	Phase II, cohort	N
